# Health Literacy and Treatment Outcomes in Multidrug-Resistant Tuberculosis: A Systematic Review

**DOI:** 10.12688/f1000research.178915.2

**Published:** 2026-10-17

**Authors:** Sri Sugiarsi, Endang Sutisna Sulaeman, Sapja Anantanyu, Anik Lestari

**Affiliations:** 1 Doctoral Program of Development Extension and Community Empowerment, Universitas Sebelas Maret, Surakarta, Central Java, 57126, Indonesia

**Keywords:** Health Literacy, Multidrug-resistant, Systematic Review, Treatment, Tuberculosis

## Abstract

**Background:**

Multidrug-resistant tuberculosis (MDR-TB) remains a serious global health threat, with treatment success rates stagnating despite advances in therapy. Health literacy, defined as the ability to access, understand, evaluate, and apply health information, may play a role as a modifiable determinant of MDR-TB treatment success.

**Methods:**

Two databases (PubMed and Scopus) were used following the PRISMA guidelines to find articles published between January 1st, 2015, and October 11st, 2025. A total of 231 records were identified and seven articles were included in this systematic review.

**Results:**

Health literacy was operationalized via patient knowledge, counseling/education, self-care behaviors, communication, and digital adherence support, with outcomes capturing adherence, completion, attitudes, self-care, and literacy levels. Synthesis suggested a consistent directionality in which literacy-oriented strategies and communication-focused approaches aligned with improved adherence and programmatic indicators, while contextual and methodological heterogeneity limited cross-study comparability.

**Conclusion:**

Available evidence supports health literacy as a promising, modifiable lever to enhance MDR-TB adherence and treatment success, particularly when addressed through multi-component, patient-centered strategies. Future work should use validated, multidomain literacy instruments and robust designs to quantify effects and guide scalable, literacy-sensitive MDR-TB programs.

## Introduction

Tuberculosis (TB) remains one of the leading causes of death from infectious disease globally, despite major advances in diagnosis and treatment. ^1^ According to the World Health Organization (WHO) Global Tuberculosis Report 2024, ^1^ an estimated 10.3 million people developed TB in 2023, with 1.3 million deaths among HIV-negative individuals and 167,000 deaths among those living with HIV. Among all forms of TB, multidrug-resistant and rifampicin-resistant tuberculosis (MDR/RR-TB) represents one of the most critical public health threats. ^2^ In 2023, approximately 410,000 people developed MDR/RR-TB, but treatment success remained at only around 50%, far below the End TB Strategy target of 90%. ^3^ The low success rate reflects the persistent challenges of long treatment duration, severe drug-related side effects, and high loss-to-follow-up rates, especially in low- and middle-income countries (LMICs) where TB burden is highest. ^4^

Treatment adherence is the cornerstone of successful MDR-TB management. ^5^ Patients are required to complete months of multidrug regimens with strict dosing schedules and frequent clinical monitoring. ^6^ Non-adherence contributes to treatment failure, relapse, and the emergence of multidrug-resistant TB (XDR-TB), which further complicates control efforts. ^7^ While structural and programmatic barriers such as drug stock-outs, limited healthcare access, and inadequate supervision are well documented, behavioral and cognitive determinants of adherence are increasingly recognized as critical but underexplored. ^8^ Patients’ understanding of their disease, their ability to follow medical instructions, and their confidence in communicating with healthcare providers are essential components of long-term treatment success. These elements are collectively influenced by health literacy. ^9^

Health literacy, as defined by the WHO and Nutbeam’s conceptual model, is the ability of individuals to access, understand, evaluate, and apply health-related information to make informed decisions regarding their health. ^1^ It encompasses three progressive domains, functional, communicative, and critical literacywhich collectively determine how effectively individuals can engage with the healthcare system. ^10^ Evidence from chronic diseases such as diabetes, hypertension, and HIV demonstrates that higher health literacy is associated with better treatment adherence, improved self-management, and reduced hospitalization rates. ^11^ Conversely, low health literacy has been linked to poor medication compliance, misunderstanding of instructions, delayed care-seeking, and increased morbidity. ^12^ In the context of TB, patients with limited literacy may struggle to comprehend complex treatment regimens, recognize side effects, or maintain consistent communication with health professionals, thereby increasing the risk of poor outcomes. ^13^

Previous studies on TB adherence have primarily focused on pharmacological, structural, or technological interventions rather than literacy-related factors. ^14^ For instance, research on directly observed therapy (DOTS) emphasized supervision and logistical support, while more recent digital adherence technologies such as electronic medication monitors and mobile phone reminders have improved monitoring capacity but do not always address the underlying issue of comprehension and empowerment. ^15^ Moreover, patient education and counseling interventions have shown promise in enhancing adherence, yet these initiatives often lack a standardized framework for assessing or improving health literacy levels. ^16^ Previous systematic reviews of drug-resistant TB have primarily synthesized interventions aimed at improving treatment adherence and retention in care, including psychosocial, educational, material, and digital adherence-support strategies. Other reviews have focused specifically on the performance of digital technologies for monitoring TB medication adherence. However, these reviews have not examined health literacy as a distinct multidimensional construct or synthesized how literacy-related capabilities, including understanding treatment information, communicating with healthcare providers, and applying health information, may influence MDR-TB treatment adherence and outcomes. A focused review is therefore needed to clarify how health literacy has been operationalized in MDR-TB research and to identify important evidence gaps for literacy-related care.

This systematic review aimed to synthesize evidence on the relationship between health literacy and MDR-TB treatment outcomes and to describe intervention approaches addressing literacy-related domains.

## Methods

### Study design

A systematic literature search was performed in PubMed and Scopus on October 11, 2025. These databases were selected a priori to provide complementary biomedical and multidisciplinary coverage of the review topic. The search was limited to these two bibliographic databases as a pragmatic scope decision and was supplemented by reference-list screening and searches of relevant grey-literature sources. Embase, Web of Science, and CINAHL were not searched. This systematic review was conducted to synthesize existing evidence on the relationship between health literacy and treatment success in patients with multidrug-resistant tuberculosis (MDR-TB) between January 1, 2015, and October 11, 2025. This review follows the Preferred Reporting Items for Systematic Reviews and Meta-Analyses (PRISMA 2020) guidelines. All identified records were imported into Zotero ( https://www.zotero.org/accessed on 11 October 2025) for reference management and automatic duplicate removal.

### Search strategy

A comprehensive literature search was performed in PubMed and Scopus databases on October 11, 2025. The search strategy combined Medical Subject Headings (MeSH) and free-text terms related to tuberculosis, health literacy, and treatment outcomes. The search key included a combination of keywords: “multidrug-resistant tuberculosis” OR “MDR-TB” OR “rifampicin-resistant tuberculosis” AND “health literacy” OR “health education” OR “health information” OR “self-care behavior” AND “treatment outcome” OR “treatment success” OR “adherence” OR “completion.”. Additional articles were identified by manually screening the reference lists of relevant studies and grey literature in WHO and World Bank repositories, as well as Google Scholar.

### Eligibility criteria

Studies were considered eligible if they investigated health literacy, patient knowledge, or educational interventions as determinants, mediators, or predictors of treatment adherence or treatment success among patients diagnosed with multidrug-resistant or rifampicin-resistant tuberculosis (MDR/RR-TB). Eligible studies included those employing quantitative, qualitative, or mixed-methods designs, published as peer-reviewed full-text articles in English between January 2015 and October 2025. Studies were excluded if they focused exclusively on drug-susceptible tuberculosis, lacked original outcome data (such as review articles, commentaries, editorials, or study protocols), or were conference abstracts without peer review.

### Study selection

Two independent reviewers screened the titles and abstracts of all retrieved records using predefined eligibility criteria in Rayyan AI software. ^17^ Data-extraction discrepancies were resolved initially through discussion and consensus between the two reviewers. When consensus could not be reached, a third reviewer independently reviewed the source article and adjudicated the discrepancy. The final agreed dataset was used for the narrative synthesis.

Data from each included study were independently extracted by two reviewers using a standardized Excel-based extraction form. Extracted variables included author, publication year, country, study population, study design, health-literacy or literacy-related exposure/intervention, evaluation method, and relevant treatment or adherence outcomes. The independently extracted data were subsequently cross-checked to identify and resolve discrepancies before synthesis ( Table 1). Data synthesis was performed through descriptive summary and thematic analysis of relationships between health literacy dimensions and MDR-TB treatment outcomes. This systematic review was not prospectively registered in PROSPERO or another systematic review registry. Although the eligibility criteria and screening procedures were defined before study selection, they were not deposited as a publicly accessible prospective protocol. The absence of prospective registration is acknowledged as a methodological limitation of this review.

**Table 1.** Main characteristics of the studies.

Author (year)	Country	Design	Study type	Duration of intervention	Evaluation	Outcome
**Charalambous et al. (2024)** ^18^	South Africa	Cluster RCT	Digital adherence system (Wisepill EvriMED + tiered feedback) vs standard care	45 months	Adherence (≥80%), clinical outcomes	Adherence improved (81% vs 51%), but no significant difference in treatment outcomes.
**Dilas et al. (2023)** ^19^	Peru	Cross-sectional	Nurse-led health education mediating social support–adherence link	10 months	SEM mediation model	Health education significantly improved adherence and mediated social support effects.
**Peresu et al. (2022)** ^20^	Eswatini	Cross-sectional	KAP of community supporters delivering MDR-TB injections	1 months	Questionnaire & observation	KAP satisfactory, but 35% held stigma; highlights need for literacy-oriented MDR-TB training.
**Thomas et al. (2021)** ^21^	India	Qualitative	Medication Event Reminder Monitor (digital pillbox for MDR-TB)	11 months	Thematic analysis (UTAUT framework)	Highly acceptable; improved organization and HCP relations; barriers: stigma, device issues.
**Horter et al. (2020)** ^22^	Uzbekistan	Qualitative	Person-centred MDR-TB care (short-course regimen)	2 months	In-depth interviews with patients & HCWs	Dialogue and education built trust and motivation; improved adherence through engagement.
**Deshmukh et al. (2018)** ^23^	India	Qualitative (Grounded Theory)	Social, family, and counselling support (PSG model)	7 months	Thematic analysis (Social Cognitive Theory)	Key adherence drivers: awareness, counselling, family/social support, nutrition.
**Leeka et al. (2025)** ^24^	Thailand	Case–control	Determinants of MDR-TB (health literacy, attitudes, self-care, income)	6 months	Multivariable logistic regression	Low HL (AOR = 2.11) independently predicted MDR-TB; poor attitude/self-care also significant.

**Note**: **RCT** = Randomized Controlled Trial; **HL** = Health Literacy **; KAP** = Knowledge, Attitude, and Practice; **MDR-TB** = Multidrug-Resistant Tuberculosis; **RR-TB** = Rifampicin-Resistant Tuberculosis; **DS-TB** = Drug-Susceptible Tuberculosis; **HCW** = Health Care Worker; **HCP** = Health Care Provider; **PSG** = Patient Support Group; **AE** = Adverse Event; **QALY** = Quality-Adjusted Life Year; **HRQoL** = Health-Related Quality of Life; **SEM** = Structural Equation Modeling; **UTAUT** = Unified Theory of Acceptance and Use of Technology; **AOR** = Adjusted Odds Ratio; **ARR** = Adjusted Risk Ratio; **LTFU** = Lost to Follow Up.

Table 1 Show Main characteristics of the included studies evaluating adherence interventions and determinants in MDR-TB and related contexts.

## Results

The systematic search yielded a total of 231 records, consisting of 42 from PubMed and 189 from Scopus. Following the removal of 68 duplicates, 163 unique records were screened for eligibility based on titles and abstracts. During this phase, 152 records were excluded for not meeting the predefined inclusion criteria due to factors such as inappropriate study design (n = 48), publication type (n = 39), study population (n = 24), or reported outcomes (n = 41). Eleven reports were sought for full-text retrieval. Two reports could not be retrieved because full-text access was unavailable, leaving nine reports for eligibility assessment. Of these, two were excluded because they did not contain a relevant literacy-related exposure or intervention. Ultimately, seven studies met the eligibility criteria and were included in the final synthesis. Ultimately, seven studies satisfied all inclusion criteria and were incorporated into the final synthesis, as illustrated in ( Figure 1).

**Figure 1.**
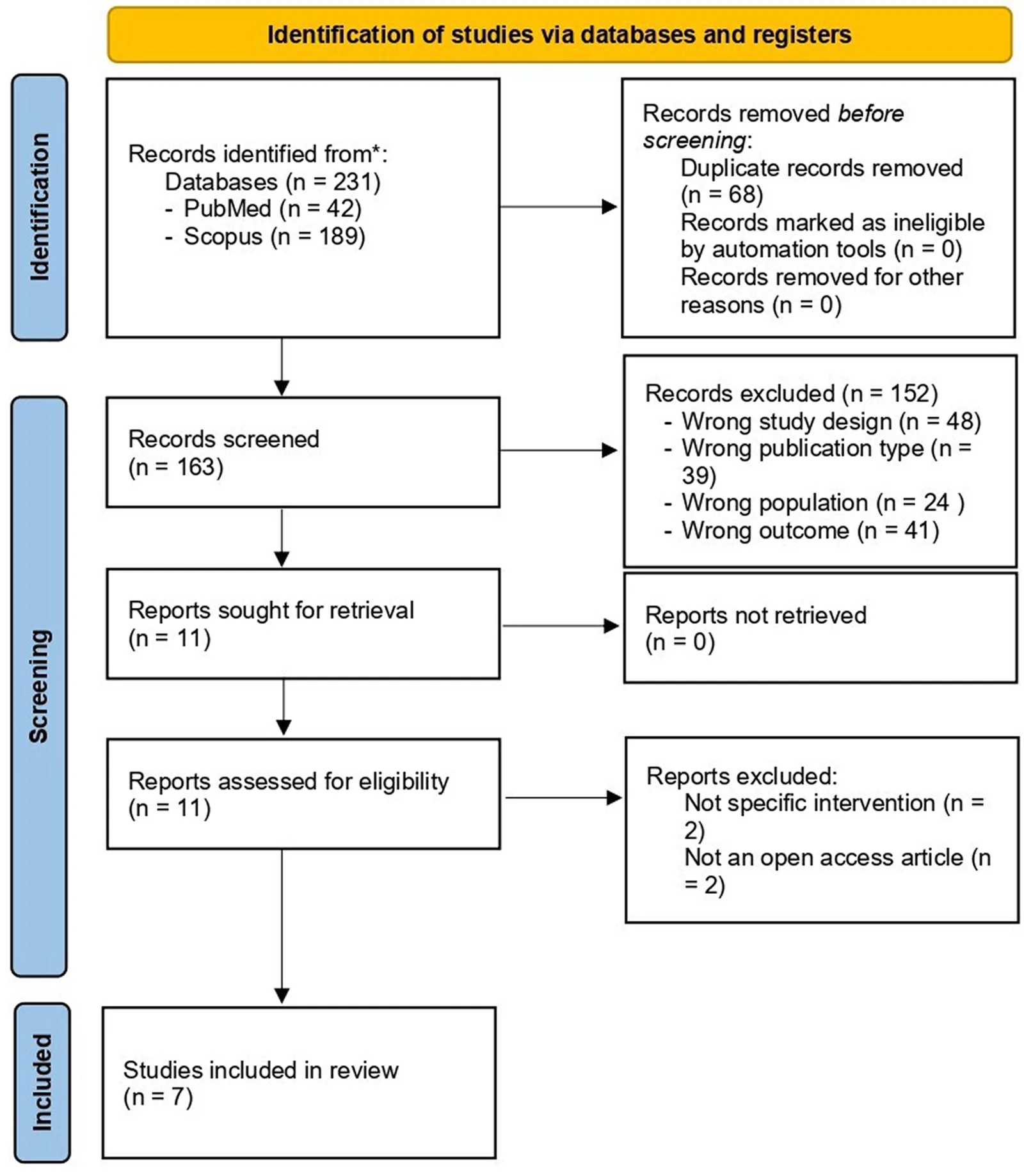
PRISMA flowchart diagram. Figure 1 illustrates the identification, screening, eligibility assessment, and inclusion of studies following the PRISMA guidelines.

### Summary of included studies

A total of seven studies published between 2018 and 2025 met the inclusion criteria and were incorporated into the final synthesis ( Table 1). These studies were conducted across six low- and middle-income countries: India (n = 2), Uzbekistan (n = 1), Thailand (n = 1), Eswatini (n = 1), Peru (n = 1), and South Africa (n = 1) (see Table 1). Collectively, they represent diverse regional contexts of multidrug-resistant tuberculosis (MDR-TB) management and various methodological approaches, including one cluster randomized controlled trial, one case–control study, two cross-sectional analytical studies, and three qualitative investigations.

The included studies differed substantially in how they addressed health literacy. Only Leeka et al. ^24^ directly assessed health literacy as an explicit patient-level construct and examined its association with MDR-TB status. Dilas et al. ^19^ evaluated nurse-delivered health education as a mediator of treatment adherence rather than directly measuring multidimensional health literacy. The remaining studies provided indirect or literacy-related evidence: Peresu et al. ^20^ assessed knowledge, attitudes, and practices among community treatment supporters; Horter et al. ^22^ and Deshmukh et al. ^23^ examined patient education, counselling, awareness, communication, and social support; and Thomas et al. ^21^ and Charalambous et al. ^18^ evaluated digital adherence-support approaches without directly measuring health literacy. These studies were therefore interpreted as evidence concerning literacy-related mechanisms or interventions rather than as direct measurements of health literacy. This distinction is particularly important because the Thailand study explicitly measured health literacy, whereas the Peru study assessed health education, not health literacy. Duration of interventions ranged from one month to forty-five months, indicating variability in the depth and sustainability of implementation across settings.

In South Africa, ^18^ a cluster randomized trial evaluated a digital adherence support system using Wisepill EvriMED with tiered feedback, demonstrating improved adherence rates compared to standard care. Cross-sectional studies in Peru ^19^ and Eswatini ^20^ investigated nurse-led health education and the knowledge, attitudes, and practices of community treatment supporters, respectively. In India ^21^ and Uzbekistan, ^22^ qualitative studies assessed patient experiences with digital pillboxes and person-centered care models, emphasizing engagement and communication in MDR-TB management. A grounded theory study ^23^ further highlighted the role of family and social support in adherence. Finally, a case–control study in Thailand ^24^ identified limited health literacy as an independent predictor of MDR-TB occurrence, underscoring literacy’s influence on patient outcomes. Overall, the included studies captured a broad spectrum of literacy-related interventions, ranging from digital tools and educational counselling to person-centered frameworks, evaluated through both behavioural and clinical indicators of adherence and treatment success.

## Discussion

Previous evidence syntheses in drug-resistant tuberculosis have primarily focused on treatment adherence, retention in care, psychosocial support, and digital adherence technologies, whereas health literacy has received comparatively limited attention as a distinct multidimensional construct. The present review therefore synthesizes the available evidence relating health literacy and literacy-related factors to treatment engagement and outcomes in MDR-TB care. Our synthesis of seven heterogeneous studies suggests that health literacy and literacy-related factors may be relevant to treatment adherence and other treatment-related outcomes in MDR-TB care; however, the strength of this relationship remains uncertain. However, it also reveals a field in its infancy, characterized by conceptual fragmentation and methodological challenges that must be addressed to translate potential into practice.

### Conceptual fragmentation and the operationalization of health literacy

A primary finding of this review is the striking conceptual inconsistency in how health literacy is operationalized across the MDR-TB literature. We observed a conceptual blurring where “health literacy” was interchangeably used to describe patient knowledge, educational interventions, self-care behaviors, and communication skills. This conflation treats health literacy as both an input and an outcome, thereby complicating the synthesis of evidence and obscuring the true mechanistic role it plays. For instance, Study in Eswatini ^20^ measured literacy via knowledge and attitudes, ^20^ study in Peru ^19^ framed it as an educational intervention mediating social support. To advance this field, it is imperative to adopt a unified theoretical framework, such as Sørensen’s Integrated Model, which distinguishes between functional (accessing and understanding information), interactive (communicating and applying information), and critical (critically appraising information for informed decision-making) health literacy dimensions. ^25^ The critical gap highlighted by our review is the complete absence of studies using validated, multidomain health literacy instruments tailored to the MDR-TB context. Without such tools, we cannot pinpoint which specific literacy dimension understanding drug side-effects (functional) or negotiating treatment with a provider (interactive) is most predictive of treatment success, thereby hindering the development of targeted interventions.

### Mechanistic pathways linking health literacy to treatment success

Beyond mere association, our synthesis elucidates the complex, multi-faceted mechanistic pathways through which health literacy influences MDR-TB outcomes. The evidence demonstrates that its role is not monolithic but context-dependent. One case-control study reported an independent association between limited health literacy and MDR-TB status; however, this finding should be interpreted as an association rather than evidence of a direct causal effect. It functions as a direct predictor, as shown study in Thailand ^24^ where low health literacy independently doubled the odds of MDR-TB. More subtly, it acts as a mediator, exemplified study in Peru, ^19^ where health literacy was the crucial cognitive and skills-based mechanism that translated external social support into internalized motivation and actionable self-care behaviors, ultimately improving adherence. Furthermore, health literacy serves as a critical enabler for digital health technologies. The studies in India ^21^ and South Africa ^18^ demonstrate that the efficacy of digital pillboxes and adherence monitors is contingent upon a patient’s baseline literacy to interpret device alerts, understand their significance, and problem-solve technical issues. This novel insight moves the field beyond a simple linear correlation and positions health literacy as a dynamic competency that interacts with and amplifies other therapeutic factors.

### The efficacy and active components of literacy-sensitive interventions

The included studies evaluated a spectrum of intervention modalities, from digital pillboxes to person-centred counselling. The available evidence suggests that both digital adherence support and interactive, dialogue-based approaches may contribute to treatment engagement; however, the heterogeneity of study designs and outcomes precludes conclusions regarding the comparative superiority of either approach. The Study in Uzbekistan ^22^ on person-centred care is paradigmatic; its effectiveness lies in its direct cultivation of interactive health literacy. By fostering open dialogue between patients and healthcare workers, ^26^ it builds trust, mutual understanding, and shared decision-making, which in turn enhances intrinsic motivation and the problem-solving skills necessary to navigate a lengthy ^27^ and arduous treatment course. ^28, 29^ A critical synthesis of these findings leads us to highlight that the most successful interventions are inherently multi-component. They do not seek to improve literacy in a vacuum but integrate literacy support within a broader framework that simultaneously addresses structural barriers such as stigma, ^23^ nutritional support, and socioeconomic constraints, creating a holistic ecosystem for patient success.

### Contextual drivers and the imperative for localized strategies

The exclusive derivation of evidence from low- and middle-income countries (LMICs) ^18, 23^ underscores the global health equity dimensions of the MDR-TB challenge and rightly focuses attention on the settings where the burden is greatest. Our review clearly indicates that local context acts as a powerful effect modifier. The impact of a health literacy intervention is not determined solely by its design but is profoundly shaped by local health system structures, cultural beliefs about illness and medication, and the pervasive level of TB-related stigma, which varied significantly between the settings in Eswatini, ^20^ Peru, ^19^ and India. ^21^ This necessitates a fundamental rejection of the “one-size-fits-all” approach. ^30^ Strategies must be locally adapted and co-designed with communities to ensure cultural resonance ^31^ and address context-specific barriers. ^24^ In this endeavour, the dual role of community health workers and peer supporters, as highlighted across multiple studies, ^32^ becomes indispensable. They act not only as literacy facilitators but also as cultural translators, effectively bridging the chasm between the formal health system and the patient’s lived experience. ^33^

Taken together, the available evidence suggests that health literacy and literacy-related factors may be potentially modifiable contributors to MDR-TB treatment engagement and adherence. The findings also indicate that education, communication, counselling, and patient-centred support may represent relevant pathways through which literacy-related needs can be addressed; however, causal relationships cannot be established from the current evidence base. However, these conclusions must be interpreted in the context of this review’s limitations. The small number of included studies (n = 7), their significant methodological heterogeneity (encompassing RCTs, cross-sectional, and qualitative designs), and the restriction to English-language publications may introduce selection bias and limit the generalizability of our findings. Furthermore, the conceptual fragmentation discussed earlier inherently constrains the robustness of any cross-study synthesis. These limitations, however, clearly chart the course for future research. There is an urgent need for prospective cohort studies that employ validated, multidimensional health literacy instruments to establish causal relationships and quantify the effect size of specific literacy dimensions. Concurrently, the field requires the development and rigorous evaluation of complex interventions specifically designed to target functional, interactive, and critical health literacy within MDR-TB care programs. Translating this evidence into practice demands that national TB programs move beyond passive information dissemination and instead integrate routine health literacy assessments and train healthcare providers in literacy-sensitive communication techniques. By doing so, we can leverage health literacy not just as a concept, but as a powerful, evidence-based tool to combat the global threat of MDR-TB. Restricting the bibliographic search to PubMed and Scopus may have reduced search sensitivity, and relevant studies indexed exclusively in Embase, Web of Science, CINAHL, or other databases may have been missed. In addition, a formal risk-of-bias assessment was not conducted; therefore, the internal validity and susceptibility to bias of individual study findings could not be systematically graded. The review was not prospectively registered in PROSPERO or another systematic review registry, which limits the ability to independently verify prespecified methods and assess potential deviations during the review process. However, the eligibility criteria and study-selection procedures were defined before screening and were applied consistently throughout the review.

## Conclusion

This systematic review suggests that health literacy may be an important and potentially modifiable factor associated with MDR-TB treatment adherence and treatment-related outcomes. Literacy-related approaches involving patient education, communication, counselling, and person-centred support appear promising; however, the current evidence is insufficient to establish causal relationships or the comparative superiority of specific interventions. Interpretation is limited by the small number of included studies, substantial methodological and conceptual heterogeneity, and inconsistent measurement of health literacy across studies. Future high-quality prospective and controlled studies using validated multidimensional health-literacy instruments are needed to clarify the magnitude and direction of these associations and to determine whether targeted literacy-related interventions improve MDR-TB treatment outcomes.

## Data Availability

All data underlying the results are available as part of the article and no additional source data are required. Extended data supporting this study are available in Figshare. ^34^ Figshare: PRISMA flowchart for “Health Literacy as a Determinant of Treatment Success in Multidrug-Resistant Tuberculosis: A Systematic Review” https://doi.org/10.6084/m9.figshare.31821010 ^34^ Data are available under the terms of the Creative Commons Zero "No rights reserved" data waiver (CC0 1.0 Public domain dedication).
