# Supplementary material for: Health Literacy and Treatment Outcomes in Multidrug-Resistant Tuberculosis: A Systematic Review
Source: F1000Res. 2026 Oct 17;15:576. Originally published 2026 Apr 19. [Version 2] doi: 10.12688/f1000research.178915.2 (PMC13760141; doi:10.12688/f1000research.178915.2)
Supplement: Peer review history

# Reviewer report: Reviewer response for version 2

**DOI:** 10.5256/f1000research.210098.r534930

**Published:** 2026-11-12

**Authors:** Miftahul Falah

**Affiliations:** 1 Universitas Muhammadiyah Tasikmalaya, West Java, Indonesia

**General assessment**

 This systematic review addresses an important and clinically relevant topic: the potential role of health literacy and literacy-related factors in MDR-TB treatment adherence and outcomes. The topic is particularly relevant in low- and middle-income countries, where the burden of drug-resistant tuberculosis remains substantial and treatment completion is challenged by long treatment regimens, adverse effects, socioeconomic barriers, and difficulties in accessing healthcare services.

 The manuscript has several strengths. The authors identify a relevant research gap, use a systematic search approach, report the study-selection process, and include evidence from different geographical and methodological contexts. The revised version also demonstrates improvement in conceptual framing and transparency compared with the earlier version. The authors have appropriately acknowledged several important limitations, including the absence of prospective registration and formal risk-of-bias assessment.

 However, despite these improvements, several methodological and interpretative concerns remain. Most importantly, the relationship between the review question, eligibility criteria, and the characteristics of the included studies needs to be more consistent. The seven included studies appear to investigate different populations, exposures, interventions, and outcomes. Some provide direct evidence on health literacy, while others address education, social support, digital adherence, or treatment-related experiences. At least one included study concerns drug-susceptible tuberculosis rather than MDR-TB, and another examines MDR-TB occurrence rather than treatment outcomes. These differences substantially affect the interpretation and generalizability of the findings.

**Major comments**

**1. Reassess the eligibility and relevance of the included studies**

 This is the most important issue requiring attention. The review aims to investigate health literacy and treatment outcomes among patients with MDR-TB. However, the included studies appear to differ considerably in their relevance to this specific question.

 For example, the Charalambous et al. study is described as involving people with drug-susceptible tuberculosis, while the Leeka et al. study investigates factors associated with developing MDR-TB. These studies do not directly represent the same population or outcome as studies examining treatment adherence and treatment success among patients diagnosed with MDR-TB.

 The authors should revisit the original eligibility criteria and provide a study-by-study justification for inclusion. A supplementary table could classify each study according to population, health literacy construct, intervention or exposure, and outcome. Studies that do not meet the predefined criteria should be excluded from the primary synthesis or clearly identified as indirect contextual evidence.

**2. Clearly distinguish direct health literacy evidence from literacy-related interventions**

 The revised manuscript has made progress in distinguishing direct health literacy measurement from broader literacy-related evidence. This distinction should be maintained consistently throughout the Results and Discussion.

 Patient knowledge, health education, counselling, communication, social support, and digital adherence support are related concepts, but they are not interchangeable with multidimensional health literacy. The authors should avoid describing all these constructs as direct measures of health literacy.

 I recommend organizing the Results into clearly defined categories:

- Direct assessment of health literacy.
- Health knowledge, education, and counselling.
- Communication, social support, and patient-centred care.
- Digital adherence interventions.

This structure would improve conceptual clarity and prevent overinterpretation of indirect evidence.

**3. Strengthen the methodological quality assessment**

 The absence of a formal risk-of-bias assessment remains a major methodological limitation. The included studies involve different research designs, and their findings should not be treated as having equivalent evidential strength.

 The authors should conduct a design-appropriate critical appraisal and present the results in a table. The Discussion should explain how methodological quality affects confidence in the findings. If a formal appraisal cannot be added, the manuscript should explicitly state that the review cannot determine the internal validity of the included studies and should substantially moderate its conclusions.

**4. Improve search strategy transparency and reproducibility**

 The authors should provide the full search strategy for PubMed and Scopus, preferably in a supplementary file. The exact search strings, search fields, filters, and dates should be reported.

 The rationale for selecting only two databases should also be strengthened. The authors should discuss the possibility that relevant studies indexed in Embase, Web of Science, CINAHL, or regional databases may have been missed.

**5. Improve the synthesis and interpretation of findings**

 The narrative synthesis is reasonable given the small number and heterogeneity of the studies. However, the authors should explain more clearly how the thematic categories were developed and how evidence from different study designs was integrated.

 The Discussion should distinguish associations from causal effects. In particular, qualitative findings and cross-sectional studies cannot establish that health literacy interventions improve treatment success. The authors should avoid implying that digital or interactive interventions are superior to other approaches without direct comparative evidence.

**6. Further refine the limitations and implications**

 The limitations section should clearly discuss the possible effects of:

- The small number of included studies.
- Heterogeneity in research designs and outcome measures.
- Inconsistent definitions and measurement of health literacy.
- Restriction to English-language publications.
- Limited database coverage.
- Exclusion of articles unavailable through institutional access.
- Absence of prospective protocol registration.
- Lack of formal risk-of-bias assessment.

The implications for clinical practice and public health policy should also be proportional to the strength of the evidence. Health literacy-sensitive care may be a promising complementary approach, but the current evidence does not establish its independent causal effect on MDR-TB treatment success.

Are the rationale for, and objectives of, the Systematic Review clearly stated?

Yes

Is the statistical analysis and its interpretation appropriate?

Partly

If this is a Living Systematic Review, is the ‘living’ method appropriate and is the search schedule clearly defined and justified? (‘Living Systematic Review’ or a variation of this term should be included in the title.)

Not applicable

Are sufficient details of the methods and analysis provided to allow replication by others?

Partly

Are the conclusions drawn adequately supported by the results presented in the review?

Partly

Reviewer Expertise:

NA

I confirm that I have read this submission and believe that I have an appropriate level of expertise to confirm that it is of an acceptable scientific standard, however I have significant reservations, as outlined above.

# Reviewer report: Reviewer response for version 2

**DOI:** 10.5256/f1000research.210098.r531020

**Published:** 2026-11-03

**Authors:** Milagros Zavaleta

**Affiliations:** 1 Research and development, BTS Consultores S.A.C., Lima ,, Peru

*The authors have updated the article with sufficient evidence addressing my observations. The additions and modifications are satisfactory.*

Are the rationale for, and objectives of, the Systematic Review clearly stated?

Partly

Is the statistical analysis and its interpretation appropriate?

Partly

If this is a Living Systematic Review, is the ‘living’ method appropriate and is the search schedule clearly defined and justified? (‘Living Systematic Review’ or a variation of this term should be included in the title.)

Not applicable

Are sufficient details of the methods and analysis provided to allow replication by others?

Partly

Are the conclusions drawn adequately supported by the results presented in the review?

Partly

Reviewer Expertise:

NA

I confirm that I have read this submission and believe that I have an appropriate level of expertise to confirm that it is of an acceptable scientific standard.

# Reviewer report: Reviewer response for version 1

**DOI:** 10.5256/f1000research.197358.r496647

**Published:** 2026-07-10

**Authors:** Frank Offei Odonkor

**Affiliations:** 1 University of Cape Coast, Cape Coast, Ghana

1. The introduction explains the importance of MDR-TB and health literacy but does not clearly identify what is missing in the current literature. Please include 2-3 sentences explicitly stating what previous systematic reviews have covered and why a review specifically on health literacy is needed.

 2. Please assess the included studies using an appropriate tool and discuss how study quality influenced the interpretation of the findings. (APPRAISE THE STUDIES)

 3. Please report whether the review protocol was developed before the study commenced. If a protocol exists, provide a reference or explain why one was not registered

 4. It is unclear whether data extraction was performed independently by two reviewers. Please clarify this to minimise the risk of extraction bias.

 5. Please can you justify why important databases such as Embase, Web of Science, or CINAHL were not included.

 6. The Results section should distinguish clearly between studies assessing health literacy directly and those evaluating educational interventions.

 7. Please describe how disagreements during data extraction were resolved. Was consensus reached through discussion, or was a third reviewer consulted?

 8. use either health literacy intervention or literacy-related intervention consistently throughout the manuscript.

 9. Statements such as "health literacy is a critical determinant" and "interactive interventions are superior" should be presented more cautiously because only seven heterogeneous studies were included, and most were observational. Consider using wording such as "the available evidence suggests" or "the findings indicate a potential association".

 10. In addition to acknowledging the small number of studies and methodological heterogeneity, in the limitation section, please discuss the potential effects of including only English-language publications and excluding non-open-access articles, as these decisions may have introduced language and publication bias.

 11. The claim, "To the best of our knowledge, this is the first systematic review...", should be supported by evidence or expressed more cautiously to avoid overstating novelty.

 12. Rather than stating that the review "establishes" health literacy as a determinant of treatment success, consider wording such as: "This review suggests that health literacy may be an important and modifiable factor associated with MDR-TB treatment success; however, further high-quality prospective studies using validated health literacy instruments are needed to confirm this relationship." This wording is more consistent with the evidence presented.

Are the rationale for, and objectives of, the Systematic Review clearly stated?

Partly

Is the statistical analysis and its interpretation appropriate?

Not applicable

If this is a Living Systematic Review, is the ‘living’ method appropriate and is the search schedule clearly defined and justified? (‘Living Systematic Review’ or a variation of this term should be included in the title.)

Not applicable

Are sufficient details of the methods and analysis provided to allow replication by others?

Partly

Are the conclusions drawn adequately supported by the results presented in the review?

Partly

Reviewer Expertise:

My research is on chronic disease self-management, health literacy, treatment adherence, and patient-centred care in nursing and public health.

I confirm that I have read this submission and believe that I have an appropriate level of expertise to confirm that it is of an acceptable scientific standard, however I have significant reservations, as outlined above.

# Response

**Published:** 2026-09-06

**Authors:** sri sugiarsi

**Affiliations:** Graduate School, Universitas Sebelas Maret, Surakarta, Central Java, Indonesia

We sincerely thank the reviewer for the careful and constructive evaluation of our manuscript. We greatly appreciate the comments regarding the conceptual framing, methodological transparency, interpretation of the evidence, and strength of the conclusions. We have carefully considered each point and revised the manuscript accordingly. In particular, we have clarified the research gap, expanded methodological reporting, distinguished direct health-literacy assessment from literacy-related interventions, standardized terminology, strengthened the limitations, and moderated statements that could imply causality or comparative effectiveness beyond the available evidence. Our point-by-point responses are provided below.

**1. Comment:**

 The introduction explains the importance of MDR-TB and health literacy but does not clearly identify what is missing in the current literature. Please include 2-3 sentences explicitly stating what previous systematic reviews have covered and why a review specifically on health literacy is needed.

**Author Response:**

 Thank you for this important comment. We agree that the research gap was not sufficiently explicit in the original Introduction. We have revised the final part of the Introduction to clarify that previous reviews of TB and drug-resistant TB have predominantly focused on treatment adherence, psychosocial support, programmatic interventions, and digital adherence technologies, whereas health literacy has received comparatively limited attention as a distinct multidimensional construct. We have therefore added a concise explanation of why a focused synthesis of health literacy and literacy-related factors in MDR-TB care is needed and refined the objective of the review accordingly.

**2. Comment:**

 Please assess the included studies using an appropriate tool and discuss how study quality influenced the interpretation of the findings. (APPRAISE THE STUDIES)

**Author Response:**

 Thank you for this important methodological comment. We agree that a formal risk-of-bias assessment would provide additional information regarding the internal validity of the included evidence. However, a formal risk-of-bias assessment was neither prespecified nor undertaken as part of the original review methodology. Because the review was designed as a descriptive and thematic synthesis of a small and highly heterogeneous body of evidence comprising randomized, observational, and qualitative designs, we elected not to introduce a new post hoc formal appraisal framework after completion of the review.

 We have instead made this methodological limitation explicit in the revised manuscript and further moderated the interpretation of the findings. Observational findings are now interpreted as associations rather than causal relationships, qualitative findings are used primarily to provide contextual and experiential insights, and claims regarding comparative intervention effectiveness have been avoided. We also explicitly acknowledge that the absence of a formal risk-of-bias assessment limits our ability to systematically judge the internal validity of individual studies and reduces certainty in the synthesized evidence.

**3. Comment:**

 Please report whether the review protocol was developed before the study commenced. If a protocol exists, provide a reference or explain why one was not registered.

**Author Response:**

 Thank you for this important comment. We agree that the protocol and registration status should be reported transparently. This systematic review was not prospectively registered in PROSPERO or another systematic review registry, and no publicly accessible prospective protocol was deposited. Nevertheless, the review question, eligibility criteria, and study-selection procedures were defined before screening and were applied consistently throughout the review.

 We have clarified this information in the Methods section and explicitly acknowledged the absence of prospective registration as a methodological limitation, because it restricts independent verification of prespecified methods and potential deviations during the review process.

**4. Comment:**

 It is unclear whether data extraction was performed independently by two reviewers. Please clarify this to minimise the risk of extraction bias.

**Author Response:**

 Thank you for highlighting this important point. We agree that the original manuscript did not provide sufficient detail regarding the data-extraction process. Data were extracted using a standardized extraction form, and during revision all extracted data were independently re-checked against the original articles by a second reviewer to minimize extraction errors and potential extraction bias. The Methods section has been revised to describe this procedure explicitly.

**5. Comment:**

 Please can you justify why important databases such as Embase, Web of Science, or CINAHL were not included.

**Author Response:**

 Thank you for this important observation. We agree that the rationale for database selection should be clearly stated. PubMed and Scopus were selected a priori because they provide complementary coverage of biomedical, clinical, public health, and multidisciplinary literature relevant to tuberculosis, health literacy, and treatment adherence. The database search was supplemented by reference-list screening and searches of relevant grey-literature sources.

 Nevertheless, we acknowledge that restricting the bibliographic search to PubMed and Scopus may have reduced search sensitivity and that relevant studies indexed exclusively in Embase, Web of Science, CINAHL, or other databases may have been missed. We have therefore clarified the database-selection rationale in the Methods and added this issue explicitly to the limitations of the review.

**6. Comment:**

 The Results section should distinguish clearly between studies assessing health literacy directly and those evaluating educational interventions.

**Author Response:**

 Thank you for this insightful comment. We agree that the previous Results section did not sufficiently distinguish direct assessment of health literacy from educational and other literacy-related interventions. We have reorganized the synthesis accordingly.

 The revised Results now distinguish studies that explicitly assessed health literacy as a patient-level construct from studies evaluating health education, counselling, communication, knowledge, social support, or digital adherence approaches. These latter studies are now described as providing indirect or literacy-related evidence rather than as direct measurements of health literacy. This distinction also prevents health literacy, patient knowledge, education, and digital adherence support from being treated as interchangeable constructs.

**7. Comment:**

 Please describe how disagreements during data extraction were resolved. Was consensus reached through discussion, or was a third reviewer consulted?

**Author Response:**

 Thank you for requesting this clarification. We agree. During the independent re-check of extracted data, any discrepancies were first discussed between the two reviewers and resolved by consensus. If consensus could not be reached, a third reviewer was available to independently examine the original article and adjudicate the discrepancy. This procedure has now been explicitly described in the Methods section.

**8. Comment:**

 Use either health literacy intervention or literacy-related intervention consistently throughout the manuscript.

**Author Response:**

 Thank you for noting this terminology inconsistency. We agree. We have standardized the terminology throughout the manuscript and now use **“literacy-related intervention(s)”** when referring to educational, counselling, communication, knowledge-based, digital, or other supportive approaches that may influence literacy-related processes.

 The term **“health literacy”** is reserved for the health-literacy construct itself or for studies that explicitly assessed health literacy. We selected “literacy-related intervention” because several included studies did not directly measure or specifically target health literacy using a validated multidimensional health-literacy framework.

**9. Comment:**

 Statements such as "health literacy is a critical determinant" and "interactive interventions are superior" should be presented more cautiously because only seven heterogeneous studies were included, and most were observational. Consider using wording such as "the available evidence suggests" or "the findings indicate a potential association".

**Author Response:**

 Thank you for this important recommendation. We fully agree. Given the small number of included studies, substantial methodological and conceptual heterogeneity, and the predominance of observational and qualitative evidence, several statements in the original manuscript were stronger than warranted by the evidence.

 We have systematically revised the Abstract, Discussion, and Conclusion to replace definitive or causal language with more cautious wording such as **“the available evidence suggests,” “may be associated with,”** and **“may represent a potentially modifiable factor.”** We have also removed statements implying that interactive interventions are superior because the available studies do not provide sufficient comparative evidence to establish superiority. The revised manuscript now consistently distinguishes association from causation and avoids claims of comparative effectiveness that are not directly supported by the included evidence.

**10. Comment:**

 In addition to acknowledging the small number of studies and methodological heterogeneity, in the limitation section, please discuss the potential effects of including only English-language publications and excluding non-open-access articles, as these decisions may have introduced language and publication bias.

**Author Response:**

 Thank you for highlighting these important potential sources of bias. We agree and have expanded the limitations accordingly. The revised manuscript now explicitly states that restricting eligibility to English-language publications may have introduced language bias and may have reduced representation of relevant evidence from non-English-speaking settings.

 We have also clarified that two potentially relevant reports could not be included because full-text access was unavailable. We acknowledge that this may have introduced availability or selection bias and reduced the comprehensiveness of the evidence base. In addition, we have revised the PRISMA reporting to distinguish reports that could not be retrieved from studies excluded after full-text eligibility assessment. These limitations are now explicitly considered when interpreting the findings.

**11. Comment:**

 The claim, "To the best of our knowledge, this is the first systematic review...", should be supported by evidence or expressed more cautiously to avoid overstating novelty.

**Author Response:**

 Thank you for this valuable observation. We agree that the original statement could overstate the novelty of the review. We have therefore removed the claim that this is the “first” systematic review.

 The revised Discussion now states more cautiously that previous evidence syntheses have primarily focused on treatment adherence, psychosocial support, retention in care, and digital adherence technologies, whereas health literacy has received comparatively limited attention as a distinct multidimensional construct in MDR-TB care. This wording more accurately positions the contribution of the present review without making an unsupported claim of absolute novelty.

**12. Comment:**

 Rather than stating that the review "establishes" health literacy as a determinant of treatment success, consider wording such as: "This review suggests that health literacy may be an important and modifiable factor associated with MDR-TB treatment success; however, further high-quality prospective studies using validated health literacy instruments are needed to confirm this relationship." This wording is more consistent with the evidence presented.

**Author Response:**

 Thank you for this constructive recommendation. We fully agree. We have substantially revised the Conclusion to ensure that it reflects the strength and limitations of the available evidence.

 The revised Conclusion now states that health literacy **may be an important and potentially modifiable factor associated with MDR-TB treatment adherence and treatment-related outcomes**, rather than stating that the review “establishes” health literacy as a determinant. We have also removed claims of superior efficacy and emphasized that the current evidence is insufficient to establish causality. Finally, we highlight the need for high-quality prospective and controlled studies using validated multidimensional health-literacy instruments to clarify the magnitude and direction of these relationships and to determine whether targeted literacy-related interventions improve MDR-TB outcomes.

 We sincerely thank the reviewer again for these thoughtful and constructive comments. We believe that the revisions have substantially improved the methodological transparency, conceptual clarity, and evidence-based interpretation of the manuscript. Latest Version can be find in this link: https://drive.google.com/drive/folders/1lcn2mkI2JU7IgzvcnIzPOHVoR6NzGl47?usp=sharing )

# Reviewer report: Reviewer response for version 1

**DOI:** 10.5256/f1000research.197358.r482828

**Published:** 2026-06-08

**Authors:** Milagros Zavaleta

**Affiliations:** 1 Research and development, BTS Consultores S.A.C., Lima ,, Peru

The review aims to summarize evidence on health literacy as a determinant of treatment success in MDR-TB and describe intervention approaches targeting literacy-related domains. This is appropriate and relevant. However, the objective should be refined into a more structured review question, ideally specifying the population, exposure or intervention, comparator where applicable, and outcomes.

 The rationale is generally clear, and the introduction explains the clinical importance of MDR-TB, the relevance of adherence, and the potential role of health literacy as a modifiable factor. The objective is also stated to summarize evidence on health literacy as a determinant of treatment success in MDR-TB and describe literacy-related intervention approaches.

 Regarding the methods, these are partially reproducible, although important details are missing. The authors report the databases searched, date range, search date, broad eligibility criteria, use of Rayyan and Zotero, and the PRISMA flow diagram on page 6. Nevertheless, the full database-specific search strategies, screening form, data extraction process, risk-of-bias assessment, certainty-of-evidence approach, and justification for excluding non-open-access articles are not sufficiently detailed. These omissions limit reproducibility.

 The use of descriptive and thematic synthesis is broadly appropriate given the small number of studies included and their heterogeneity. The included studies vary substantially in design, intervention type, outcome measures, and analytical methods, as shown in Table 1 on page 5. Nonetheless, the review does not adequately justify the absence of meta-analysis, does not provide a structured heterogeneity assessment, and does not include risk-of-bias or certainty-of-evidence assessment. Some interpretations are stronger than the underlying statistical evidence supports.

 It is suggested that the authors make the conclusions more cautious. The results support the view that health literacy-related factors may be relevant to MDR-TB adherence and patient engagement. That said, the evidence base consists of only seven heterogeneous studies, including observational and qualitative designs, and health literacy is inconsistently defined across studies. Claims that health literacy is a “critical determinant” or that certain interventions have superior efficacy are not fully demonstrated by the presented evidence.

 The conclusion should emphasize association and potential importance rather than definitive causal or effectiveness claims.

Are the rationale for, and objectives of, the Systematic Review clearly stated?

Partly

Is the statistical analysis and its interpretation appropriate?

Partly

If this is a Living Systematic Review, is the ‘living’ method appropriate and is the search schedule clearly defined and justified? (‘Living Systematic Review’ or a variation of this term should be included in the title.)

Not applicable

Are sufficient details of the methods and analysis provided to allow replication by others?

Partly

Are the conclusions drawn adequately supported by the results presented in the review?

Partly

Reviewer Expertise:

Molecular biology, MDR-TB genotyping, development and research for diagnostic tools

I confirm that I have read this submission and believe that I have an appropriate level of expertise to confirm that it is of an acceptable scientific standard, however I have significant reservations, as outlined above.

# Response

**Published:** 2026-06-30

**Authors:** sri sugiarsi

**Affiliations:** Graduate School, Universitas Sebelas Maret, Surakarta, Central Java, Indonesia

We would like to express our sincere gratitude to the reviewer for the meticulous and highly constructive feedback. We deeply appreciate the time and expertise invested in evaluating our manuscript. The reviewer has raised several crucial methodological and conceptual points that have helped us sharpen the clarity and scientific rigor of our systematic review.

 We have carefully addressed each comment below. Since we are responding via an open-review format, we have detailed our proposed revisions and methodological clarifications extensively in this response. We are fully committed to incorporating all these changes in the final revised version of the manuscript.

 1. We completely agree with this essential suggestion. In our revised manuscript, we have reformulated the objective into a structured PICo framework (Population, phenomenon of Interest, and Context), which is the most appropriate approach for mixed-methods and qualitative evidence synthesis.

**We will revise the objective section to read:**

***"This systematic review was guided by the following structured question: In patients with Multidrug-Resistant Tuberculosis (Population), what is the role of health literacy or literacy-oriented interventions (phenomenon of Interest) within MDR-TB treatment programs (Context) in determining treatment adherence and success? Specifically, we aimed to: (1) synthesize the association between health literacy domains and treatment outcomes, and (2) describe the characteristics of interventions designed to address literacy-related barriers in MDR-TB care."***

 2. The reviewer has rightly identified a critical gap. To ensure full transparency and reproducibility, we have elaborated on each of these missing components in our revised methodology. **We provide the detailed clarifications below, which we will add to the Methods section:**

**- Full Search Strategy: We acknowledge the omission. We have now compiled the complete Boolean search syntax for both PubMed and Scopus (including all MeSH terms and free-text words). Due to space constraints, we will place this detailed syntax in a Supplementary File. In the main text, we will specify the core search blocks: ("multidrug-resistant tuberculosis" OR "MDR-TB") AND ("health literacy" OR "health education" OR "self-care") AND ("treatment outcome" OR "adherence").**

**- Screening and Data Extraction: We have explicitly detailed that two independent reviewers conducted the screening using Rayyan AI. Discrepancies were resolved by a third reviewer. For data extraction, we utilized a standardized Excel form that was piloted on two random studies before full extraction. This form captured: author, year, country, study design, intervention type, evaluation method, and primary outcomes.**

**- Justification for Excluding Non-Open-Access Articles: We thank the reviewer for pointing this out. We excluded the two non-open-access articles strictly due to institutional access limitations and not due to scientific quality. We fully acknowledge this as a potential selection bias. We have now explicitly stated this limitation in the Discussion section: "The exclusion of non-open-access articles due to institutional access constraints may introduce selection bias and limit the comprehensiveness of our synthesis."**

**3. This is a very fair and constructive critique. We have substantially revised this section:**

**- Justification for No Meta-Analysis: We have added a clear rationale in the Methods. We state that a statistical meta-analysis was deemed inappropriate and methodologically unsound due to extreme clinical, methodological, and statistical heterogeneity. The included studies varied not just in design (RCTs vs. cross-sectional vs. qualitative), but also in how "health literacy" was defined (e.g., knowledge, communication, digital support), how "adherence" was measured (e.g., self-report vs. digital monitors), and in the duration of follow-up (ranging from 1 to 45 months). Pooling these disparate effect sizes would produce a meaningless summary estimate. Therefore, we chose a narrative thematic synthesis, which is the recommended approach by the Cochrane Handbook for such heterogeneous evidence.**

**- Structured Heterogeneity Assessment: We have now included a dedicated paragraph in the Results that explicitly describes the sources of heterogeneity: (1) geographical diversity (six LMICs with different health system structures), (2) conceptual diversity (different operationalizations of health literacy), and (3) outcome diversity (adherence rates vs. odds ratios vs. qualitative perceptions).**

**- Toning Down Interpretations: We absolutely agree with the reviewer. We have carefully revised our language throughout the manuscript. We have removed definitive causal language such as "critical determinant" and replaced it with "potentially important modifiable factor" or "associated with". We have also removed "superior efficacy" and replaced it with "showed promise" or "suggested improved outcomes in the included studies". We have explicitly stated that the evidence is associative, not causal, due to the predominance of observational designs.**

 4. We wholeheartedly accept this advice and have undertaken a thorough revision of our Abstract, Discussion, and Conclusion sections to ensure they align precisely with the strength of the evidence.

**In the revised manuscript, we will replace the current conclusion with the following cautious and evidence-aligned text:**

**"This systematic review suggests that health literacy is a potentially important modifiable factor associated with improved MDR-TB adherence and treatment success. The synthesized evidence indicates that interactive, multi-component, and person-centred interventions show promise in enhancing patient engagement. However, these findings must be interpreted with caution. The evidence base is limited by a small number of studies (n=7), substantial methodological heterogeneity, inconsistent definitions of health literacy, and a lack of robust controlled designs. Consequently, the current evidence supports an association rather than a definitive causal relationship. Future research urgently needs to employ validated, multidomain health literacy instruments and prospective cohort or randomized designs to quantify the true effect size and guide policy. Until then, health literacy should be considered a complementary strategy to, rather than a replacement for, structural and pharmacological interventions."**

 We are deeply grateful to the reviewer for pushing us to improve the academic rigor of our work. We believe that by incorporating these clarifications, our systematic review now meets the highest standards of reproducibility and intellectual honesty. We hope the reviewer finds our responses satisfactory, and we look forward to the opportunity to publish our revised manuscript.
